# Ultrafast dynamic contrast-enhanced breast MRI with quantitative perfusion parameters in differentiating breast cancer: a study focusing on triple-negative and HER2 positive breast cancer

**DOI:** 10.3389/fonc.2024.1457918

**Published:** 2025-01-07

**Authors:** Guo Haodong, Zhu Jianguo, Dmytro Pylypenko, Dou Weiqiang, Su Sheng, Xu Jie, Li Haige

**Affiliations:** ^1^ Department of Radiology, the Second Affiliated Hospital of Nanjing Medical University, Nanjing, China; ^2^ Department of Advanced Applications, General Electric Healthcare, Beijing, China

**Keywords:** differential subsampling with cartesian ordering contrast-enhanced MRI, HER2 positive breast cancer, triple-negative breast cancer, kep, HER2

## Abstract

**Background:**

In the realm of breast cancer diagnosis and treatment, accurately discerning molecular subtypes is of paramount importance, especially when aiming to avoid invasive tests. The updated guidelines for diagnosing and treating HER2 positive advanced breast cancer, as presented at the 2021 National Breast Cancer Conference and the Annual Meeting of the Chinese Society of Clinical Oncology, highlight the significance of this approach. A new generation of drug-antibody combinations has emerged, expanding the array of treatment options for HER2 positive advanced breast cancer and significantly improving patient survival rates. Triple-negative breast cancer (TNBC), on the other hand, may indicate survival outcomes following multi-agent adjuvant chemotherapy. DISCO is a more recent DCE MRI technique that has achieved high spatial and temporal resolution and minimized image artifacts in cases like malignant focal liver lesions, enhanced focal breast lesions, and intracranial aneurysms.

**Objective:**

To employ the method mentioned above to differentiate between triple-negative and non-triple-negative as well as HER2 positive and HER2 negative cancer lesions, and to assess the value of quantitative and semi-quantitative parameters in molecularly typing breast cancer.

**Methods:**

All participants were scanned with a 3.0-T MR scanner (GE SIGNA™ Premier) using a 16-channel phased-array body coil. Each participant underwent a DISCO DCE-MRI with a scan time of approximately 1 minute and 40 seconds. The ROIs were outlined with the GenIQ software, avoiding regions with blood vessels, susceptibility artifacts, hemorrhage, and necrosis. We evaluated four quantitative parameters (K^trans^, k_ep_, v_e_, v_p_) and four semi-quantitative parameters (TTP, MAX Conc, AUC, MAX Slope). The carcinomas were segregated into respective subgroups (HER2+ vs HER2-, TNBC vs non-TNBC, HER2+ vs TNBC) and we compared the eight parameters across these groups. The AUC of the models was compared using DeLong’s test as per the ROC analysis.

**Results:**

We analyzed a total of 96 female patients, revealing significant differences in the semi-quantitative parameters such as TTP, MAX Conc, AUC, and MAX Slope among different groups. HER2-positive versus HER2-negative exhibited significant differences in quantitative parameters (Ktrans: 0.22 min-1 vs. 0.43 min-1, kep: 0.11 min-1 vs. 0.35 min-1, vp: 0.01 vs. 0.04, all P < 0.05). TNBC versus non-TNBC revealed statistical variations in quantitative parameters (Ktrans: 1.03 min-1 vs. 0.15 min-1, kep: 0.61 min-1 vs. 0.19 min-1, vp: 0.18 vs. 0.01, all P < 0.05). Additionally, HER2-positive compared to TNBC demonstrated significant differences in quantitative parameters (Ktrans: 0.22 min-1 vs. 1.03 min-1, kep: 0.11 min-1 vs. 0.61 min-1, vp: 0.01 vs. 0.18, all P < 0.05). As per ROC analysis, Ktrans, kep, vp, TTP, and MAX Conc effectively differentiated TNBC from non-TNBC, with TTP being the strongest determinant for TNBC. Furthermore, these parameters successfully distinguished between HER2 positive and HER2 negative, with kep being particularly effective in identifying HER2. Importantly, Ktrans, kep, vp, TTP, and MAX Conc were effective in discriminating HER2 positive from TNBC, with kep and TTP exhibiting notable efficacy in this context.

**Conclusion:**

Our study suggests that DISCO DCE-MRI derived parameters could serve as reliable quantitative biomarkers for differentiating between TNBC and HER2 positive breast cancer.

## Introduction

Breast cancer is the principal cause of cancer-related fatalities in women, comprising 30% of all new cancer cases and contributing to 15% of women’s cancer deaths. The prognosis and treatment can differ dramatically for patients with distinct forms of breast cancer (1). As stated in the 2013 St. Gallen International Breast Cancer Conference, breast cancer can be differentiated based on molecular subtypes such as luminal A, which is characterized by estrogen receptor (ER) positivity and progesterone receptor (PR) positivity (where ER and PR status are jointly assessed as hormone receptor [HR] status), and human epidermal growth factor receptor 2 (HER 2) negativity. Luminal B subtype displays HR+, HER2+. Further subtypes include HER 2 + (overexpression of HER2 along with ER/PR+), basal-like Triple-negative breast cancer (TNBC, defined by ER−, PR−, HER2−), among other specific subtypes (2). The luminal A and B variants predict a 10-year outcome regardless of systemic treatments, and a persistent risk of distant relapse post 5 years of hormone therapy (3). Contrary to these two, HER2+ and TNBC are known to be clinically more aggressive and carry a worse prognosis (4). A new era of antibody-linked drugs has shown to broaden treatment options for HER2+ patients, substantially lengthening their life span (5). However, TNBC doesn’t respond to hormone therapy or targeted molecular therapy due to its unique molecular characteristics (6). Chemotherapy typically serves as the primary systemic treatment, but conventional adjuvant chemotherapy after surgery has limited effectiveness (7). Identifying the distinction between HER2+ and TNBC is crucial for determining appropriate treatment strategies. Histopathological categorization through biopsy tests currently serves as the gold standard for identifying breast cancer subtypes, yet their invasive nature and potential for heterogeneous lesions can restrict their clinical use (8). Therefore, a noninvasive, effective method for evaluating different breast cancer types, particularly HER2+ and TNBC, is desirable.

Magnetic resonance imaging (MRI) is widely used in the clinical diagnosis of breast diseases due to its high resolution, excellent tissue contrast, and multi-functionality (8–10). Several MRI techniques have been developed for breast cancer detection, such as diffusion-weighted imaging (DWI), quantitative MRI, chemical exchange saturation transfer MRI, and dynamic contrast-enhanced (DCE) imaging. While these have shown promising results, they’ve mostly been focused on differentiating between benign and malignant breast tumors, or evaluating treatment response to neoadjuvant chemotherapy (NAC). Limited work has been done to distinguish pathological molecular breast cancer subtypes (10).

The recently developed Differential subsampling with cartesian ordering (DISCO) contrast-enhanced MRI is a promising DCE MRI technique (10). By utilizing a pseudo-random variable density k-space segmentation and k-space shared reconstruction scheme, DISCO provides higher spatial and temporal resolution in a single scan, allowing it to deliver a more accurate measurement of the late arterial phase in dynamic liver measurement compared to traditional DCE (11). Moreover, initial studies have employed Ultrafast DCE in diagnosing breast cancers (12). The resultant semi-quantitative parameter of max slope was found to correlate with TNBC or HER2+ breast cancer, although the study was limited by lesion size and subtype, and only semi-quantitative parameters were investigated. These promising findings suggest further exploration of ultrafast DISCO imaging’s potential to deliver more reliable performance using both semi-quantitative and quantitative parameters derived from a kinetic model.

The main objective of this study, therefore, is to examine the feasibility of using ultrafast temporal resolution DISCO imaging to diagnose HER2 + and TNBC breast cancers, utilizing both quantitative and semi-quantitative perfusion parameters.

## Materials and methods

### Patients

Our study received approval from the institutional review board and was exempted from the requirement for written informed consent. We carried out the study adhering to the regulations stipulated by the Health Insurance Portability and Accountability Act

**Figure 1:**
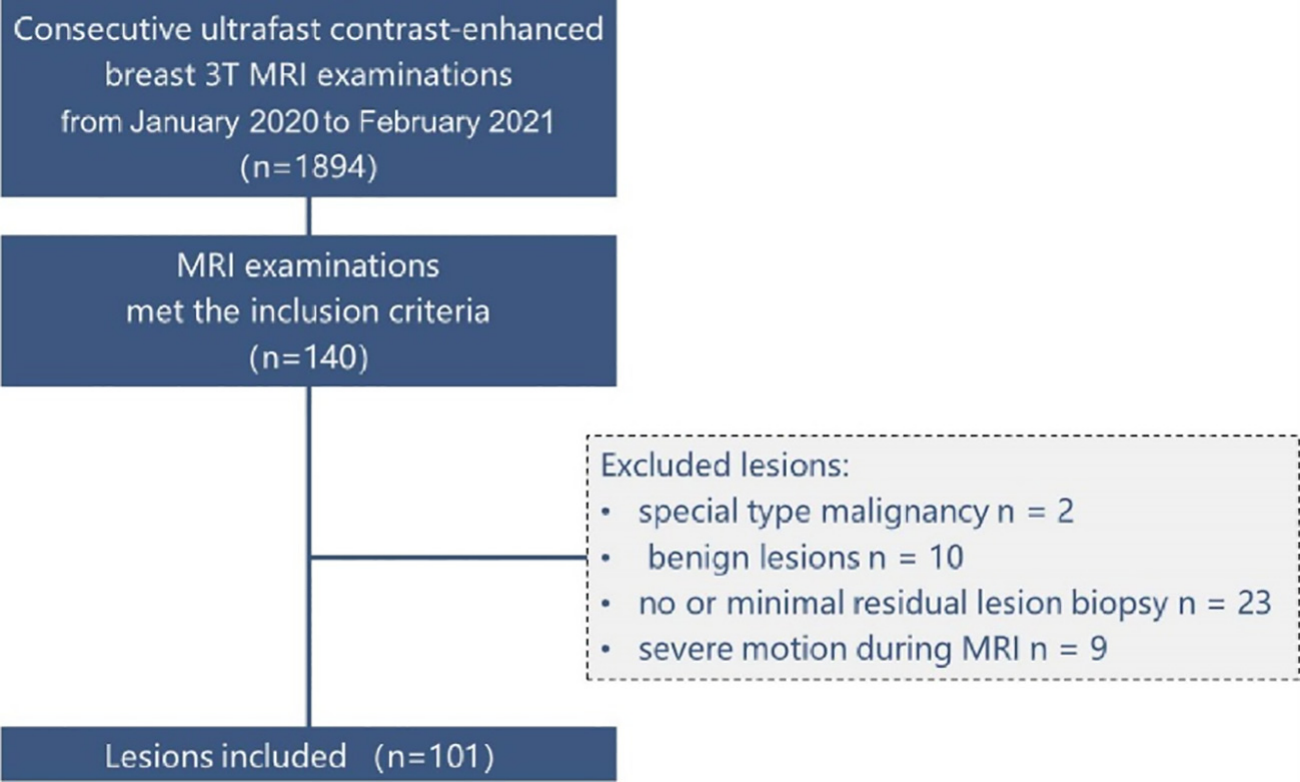
Patients selection chart.

Between January 2020 and February 2021, our institution implemented the DISCO protocol and utilized it for clinical evaluations including BI-RADS classification. We reviewed our institutional electronic health records during this timeframe to find examinations that satisfied the following eligibility criteria: (1) utilization of a hybrid ultrafast DISCO protocol on a singular 3T scanner equipped with a 16-channel breast coil, (2) depiction of breast abnormalities confirmed pathologically, (3) categorization as BI-RADS MRI 4–6, and (4) exclusion of examinations performed for post-therapeutic assessments after surgery or chemotherapy. Out of 1894 continuous ultrafast contrast-enhanced breast 3T MRI assessments, which included both screening and diagnostic examinations, 140 complied with the inclusion criteria. We incorporated all pathologically confirmed breast abnormalities exhibited on these examinations, barring the following exceptions: anomalies pathologically confirmed as unique type malignancies (n = 2, malignant phyllodes tumor), anomalies pathologically confirmed as benign abnormalities (n = 10), abnormalities with no or minor remaining enhancement challenging to differentiate from post-biopsy alterations (n = 23; invasive carcinoma, 19; ductal carcinoma *in situ* [DCIS], 4), and anomalies associated with severe patient motion during MRI scanning, unresolvable via motion correction techniques (n = 9; invasive carcinoma, 7; DCIS, 2). Totally, we included 96 examinations revealing 101 pathologically confirmed breast cancer abnormalities in this study. The detailed patient selection process is illustrated in **Figure 1**.

### MRI experiments

A 3.0T MRI scanner was used for all the MRI experiments (GE SIGNA™ Premier) with a 16-channel breast coil at three time points. The acquisition parameters were established as follows: In the case of ultrafast DCE-MRI, we applied a 3D fat-suppressed T1-weighted differential sub-sampling with Cartesian ordering (DISCO) sequence. This sequence involved a TR of 3.8ms, TE1 and TE2 set at 1.1/2.2 ms, a flip angle of 12°, a field of view measuring 34 cm × 34 cm, an acquired matrix of 212 × 212, an in-plane spatial resolution of 1.6 × 1.6 mm, a thickness of 1.6 mm, and 166 or more slices for comprehensive breast coverage. The temporal resolution was set between 2.7–4.6s/phase, starting from the contrast injection, a total of 26 phases, with a scan duration of approximately 1 minute 20 seconds to 1 minute 40 seconds. Following the ultra-early scan, the standard DCE-MRI with a time resolution of about 75, consisting of 4 phases, with a scan duration of approximately 5 minutes. We used a 3D fat-suppressed T1-weighted volume imaging breast assessment (VIBRANT) sequence with a TR/TE of 7.9/4.3, a flip angle of 12°, a field of view measuring 34 cm × 34 cm, an acquired matrix of 300 × 300, an in-plane spatial resolution of 1.1 × 1.1 mm, an adiabatic fat suppression technique, a variable number of slices set at 190 depending on the required coverage, a bandwidth at ± 62.5 kHz, an axial orientation.

Image analysis: Two radiologists (Guo and Li, with 5 and more than 20 years of experience in breast MRI, respectively) collaboratively analyzed the first post-contrast standard DCE-MR images for all abnormalities. They classified the BI-RADS MRI lesion types into mass and non-mass enhancement (NME) categories, and achieved a consensus. During this process, they remained unaware of the respective pathological findings.

**Figure 2:**
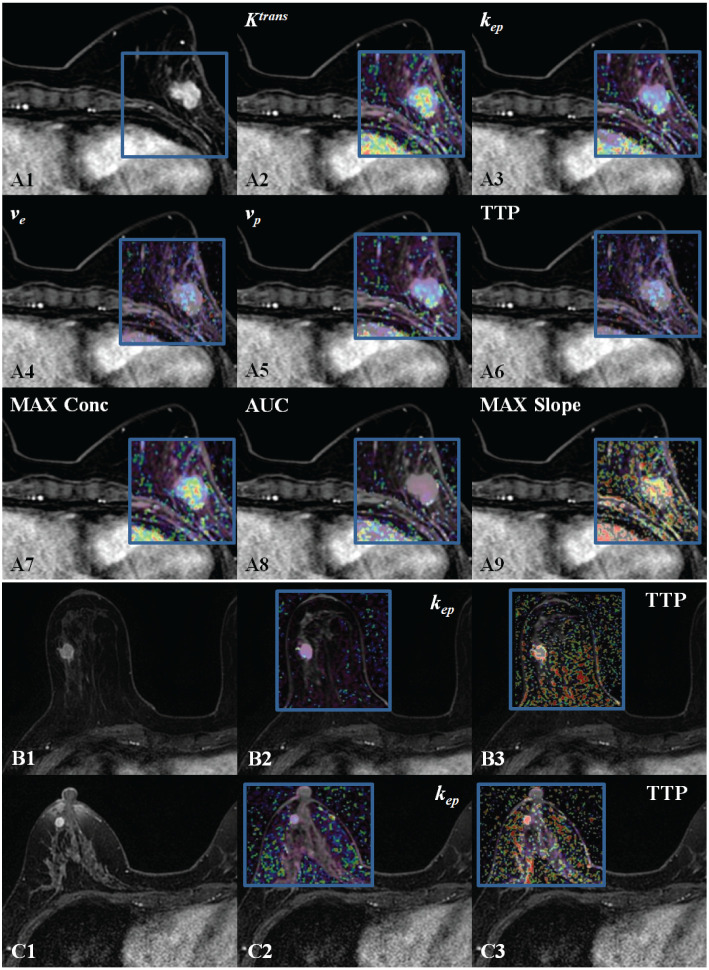
DISCO CE-MRI original map and pseudo color map generated by the tumor area. Clearly enhanced lesions can be seen within the gland of the breasts. **(A1)**, female with breast cancer (HER 2+). **(A2–A9)** are the pseudo color images of *K^trans^*, *k_ep_*, *v_e_*, *v_p_*,TTP, MAX Conc, AUC, and MAX Slope; **(B1)**, female with TNBC. **(B2, B3)**( are the pseudo color images of kep and TTP. **(C1)**, female with breast cancer (neither HER 2+ nor TNBC). **(C2, C3)** are the pseudo color images of *kep* and TTP.

The GenIQ software (13) (advantage workstation server 3.2 and advantage workstation volume share 7, AW 4.7 GE Healthcare) was utilized by the radiologists to evaluate the lesions. The semi-automatic volumetric region of interest (ROI) was created at it depicted in **Figure 2**. Initially, the radiologists manually defined the ROI on a single slice. Following this, the software generated a volumetric ROI drawing on the single slice ROI as a reference. If needed, this volumetric ROI was manually adjusted by the radiologists. The radiologists scrutinized the time-signal intensity curves of the segmented volumes along with ultrafast DCE-MR images. In case patient motion artifacts were evident in the images, a localized, rigid motion correction method was put into effect. Finally, they procured the quantitative and semi-quantitative parameters of the lesion. There were four quantitative parameters involved: the transfer constant (Ktrans), the rate constant from the interstitium to the plasma (kep), the fractional volume of the extracellular space (ve), and the volume fraction of blood plasma (vp). Additionally, four semi-quantitative parameters were calculated: time to peak (TTP), maximum concentration (MAX Conc), area under the curve (AUC), and maximum slope (MAX Slope). The following are the formulas and clarifications for these parameters:

*K^trans^* represents the volume transfer rate of contrast agent from the blood plasma to the extravascular extracellular space (EES). It can be calculated using the Tofts model:


$$K^{trans}=\left(Ctissue/Cp\right)/\left(1/Vp\right)$$

where:

Ctissue is the contrast agent concentration in the tissue of interest over time.

Cp is the arterial input function (AIF) or contrast agent concentration in the blood plasma over time.

*v_p_* is the plasma volume fraction, which represents the proportion of blood plasma volume in the tissue of interest.

*k_ep_* represents the rate constant for transfer of contrast agent from the EES back to the blood plasma. It can be calculated as:


$$k_{ep}=K^{trans}/v_{e}$$

where:

*v_e_* is the extravascular extracellular volume fraction, which represents the proportion of EES volume in the tissue of interest.

*v_e_* represents the volume fraction of EES in the tissue of interest.

*v_p_* represents the proportion of blood plasma volume in the tissue of interest.

TTP represents the time it takes for the contrast agent concentration in the tissue of interest to reach its maximum value. It can be determined by analyzing the time-intensity curve.

MAX Conc represents the peak concentration of the contrast agent in the tissue of interest. It can be obtained from the time-intensity curve.

MAX Slope represents the steepest slope of the time-intensity curve, indicating the rate of contrast agent uptake or washout in the tissue of interest. It can be determined by analyzing the time-intensity curve.

### Statistic analysis

All data analysis was executed using SPSS 23.0 and MedCalc 16.0 statistical software. The analyses were exploratory in nature, and adjustments were not made for multiple comparisons. Statistical significance was achieved when P values were less than 0.05. The reliability of the quantitative and semi-quantitative parameters between two readers was tested using the intraclass correlation coefficient for a random sample of 24 lesions from the total cohort. Radiologist 2 independently conducted segmentation and parameter calculation following the same methodology as Radiologist 1. The interpretation of the intraclass correlation coefficient between the two radiologists was classified as follows: a score between 0.75 and 1.00 indicated excellent agreement, 0.60 to 0.74 showed good agreement, 0.40 to 0.59 represented fair agreement, and anything below 0.40 pointed to poor agreement (14).

Carcinomas were categorized into different subgroups (HER2+ vs HER2-, TNBC vs non-TNBC, HER2+ vs TNBC) for comparison of quantitative and semi-quantitative parameters. We employed the Kolmogorov-Smirnov test. Data that followed a normal distribution were presented as mean ± standard deviation, while data that did not follow a normal distribution were expressed as median values (interquartile range). When the data were normally distributed with equal variances between groups (confirmed by the Levene test), analysis of variance (ANOVA) was applied. If not, the Kruskal Wallis test was utilized. For cancer traits that demonstrated significant differences, exploratory multivariate logistic regression modeling was also performed. The DeLong’s test was used to compare the area under the ROC curve (AUC) of the models.

## Results

### Characteristics of the patients

Overall, 96 female patients (average age 52 years, standard deviation 9 years, age range 32 to 76 years) with 101 pathologically confirmed cases of breast cancer lesions were analyzed. Out of these 96 patients, 91 had one lesion, and 5 had two. The patient details such as age, BI-RADS classification, menopausal status, previous breast cancer history, and family history of breast or ovarian cancer are outlined in **Table 1**.

**Table 1:** Detailed demographics of patients.

Patient characteristics	Total (n = 101)
Age	Age
Mean ± SD	52 ± 9 years
Range	32–76 years
Menopausal status	
Pre-menopause	62 (61)
Post-menopause	39 (39)
BI-RADS	
Category 6	1(1)
Category 5	7 (7)
Category 4	93 (92)
Past history of breast cancer	
Positive	1 (1)
Negative	101 (99)
Family history of breast cancer	
Positive	58 (57)
Negative	42 (42)
Not available	1 (1)
Family history of ovarian cancer	
Positive	2 (2)
Negative	97 (96)
Not available	2 (2)

Unless otherwise specified, data represent the number of patients and data in parentheses are percentages.

SD, standard deviation.

### Inter-observer agreement analysis

Excellent inter-observer agreement of DISCO CE-MRI derived semi-quantitative and quantitative parameter measurements between two observers was validated with high ICC coefficients ranging from 0.895 to 0.955 (**Table 2**).

**Table 2:** Inter-observer agreement analysis of DISCO parameter measurements of breast lesions between two radiologists.

Parameter	ICC	95% CI	*P* value
*K^trans^* (min^-1^)	0.925	0.910~0.942	0.001
*k_ep_* (min^-1^)	0.895	0.868~0.941	0.003
*v_e_*	0.901	0.889~0.928	<0.001
*v_p_*	0.939	0.926~0.966	<0.001
TTP (s)	0.955	0.937~0.979	<0.001
MAX Conc	0.924	0.918~0.952	0.001
AUC	0.951	0.933~0.987	<0.001
MAX Slope (s^-1^)	0.926	0.892~0.949	<0.001

ICC, intraclass correlation coefficient.

95% CI, 95% confidence interval.

### Comparison of perfusion parameters in HER2 positive vs HER2 negative, TNBC vs non-TNBC, and HER2 positive vs TNBC breast cancer groups

Semi-quantitative parameters of TTP, MAX Conc, AUC, and MAX Slope were found to have significant differences between the following groups: HER2 positive vs. HER2 negative (TTP: 0.91 s vs. 1.57 s, MAX Conc: 0.50 vs. 0.90, AUC: 2.12 vs. 3.29, MAX Slope: 0.40 s^-1^ vs. 1.57 s^-1^, all *P <*0.05), TNBC vs. non-TNBC (TTP: 2.03 s vs. 1.28 s, MAX Conc: 0.77 vs. 1.76, AUC: 8.04 vs. 2.56, MAX Slope: 3.97 s^-1^ vs. 0.75 s^-1^, all *P <*0.05), and HER2 positive vs. TNBC (TTP: 0.91 s vs. 2.03 s, MAX Conc: 0.50 vs. 0.77, AUC: 2.12 vs. 8.04, MAX Slope: 0.40 s^-1^ vs. 3.97 s^-1^, all *P <*0.05).

Additionally, for quantitative parameters, *K^trans^*, *k_ep_*, and *v_p_* showed statistical differences between the following groups: HER2 positive vs HER2 negative (*K^trans^*: 0.22 min^-1^ vs. 0.43 min^-1^, *k_ep_*: 0.11 min^-1^ vs. 0.35 min^-1^, *v_p_*: 0.01 vs. 0.04, all *P <*0.05), TNBC vs non-TNBC (*K^trans^*: 1.03 min^-1^ vs. 0.15 min^-1^, *k_ep_*: 0.61 min^-1^ vs. 0.19 min^-1^, *v_p_*: 0.18 vs. 0.01, all *P <*0.05), and HER2 positive vs TNBC (*K^trans^*: 0.22 min-1 vs. 1.03 min-1, *k_ep_*: 0.11 min-1 vs. 0.61 min-1, *v_p_*: 0.01 vs. 0.18, all *P <*0.05).

### Diagnostic efficacy analysis of each perfusion parameters in distinguishing breast cancers with different subtypes

With ROC analysis, *K^trans^*, *k_ep_*, *v_p_*, TTP, and MAX Conc were able to effectively distinguish TNBC from non-TNBC, with high AUCs ranging from 0.667 to 0.928. Further with De-long test, TTP showed the most robust performance in identifying TNBC, with an AUC of 0.928(*P <*0.002).

We also observed that *K^trans^*, *k_ep_*, *v_p_*, TTP, and MAX Conc were able to distinguish HER2 positive from HER2 negative with high AUCs ranging from 0.654 to 0.832.*k_ep_* was particularly effective in diagnosing HER2, with an AUC of 0.832 as determined by the Delong test (*P <*0.005).

**Figure 3:**
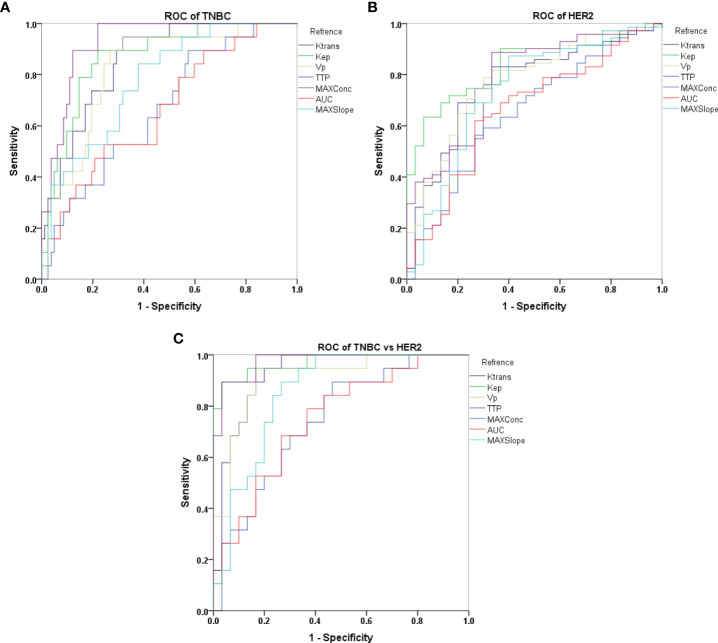
Receiver operating characteristic (ROC) curves representing different semi-quantitative and quantitative perfusion parameters derived from DISCO CE-MRI in differentiating TNBC vs non-TNBC **(A)**, HER2 vs non-HER2 **(B)** and TNBC vs HER2 **(C)**.

Furthermore, *K^trans^*, *k_ep_*, *v_p_*, TTP were able to differentiate HER2 positive from TNBC, with high AUCs ranging from 0.853 to 0.975. *k_ep_* and TTP were especially effective in this regard, with the highest AUC of 0.970 and 0.975 according to the DeLong test(*P <*0.020) (**Tables 3**, **4**; **Figure 3**).

**Table 3:** Diagnostic efficacy analysis of each perfusion parameters in breast cancer with different subtypes.

Groups	Parameters	AUC of ROC	Sensitivity	Specificity	PPV	NPV	*P* value
TNBC	*K^trans^*	0.856	18/19	56/82	18/44	56/57	<0.001
19 vs 82	*k_ep_*	0.87	17/19	64/82	17/35	64/66	<0.001
	*v_p_*	0.832	16/19	62/82	16/36	62/65	<0.001
	TTP	0.928	19/19	64/82	19/37	64/64	<0.001
	MAXConc	0.668	17/19	32/82	17/67	32/34	0.023
	AUC	0.667	10/19	62/82	10/30	62/71	0.024
	MAXSlope	0.78	16/19	51/82	16/47	51/54	<0.001
HER2	*K^trans^*	0.763	20/30	59/71	20/22	59/69	<0.001
30 vs 71	*k_ep_*	0.832	7/30	47/71	7/31	47/70	<0.001
	*v_p_*	0.775	20/30	56/71	20/35	56/66	<0.001
	TTP	0.781	19/30	63/71	19/27	63/74	<0.001
	MAXConc	0.654	22/30	40/71	22/53	40/48	0.015
	AUC	0.662	22/30	44/71	22/49	44/52	0.011
	MAXSlope	0.738	18/30	62/71	18/27	62/74	<0.001
HER2	*K^trans^*	0.926	24/30	18/19	24/25	18/24	<0.001
vs TNBC	*k_ep_*	0.970	28/30	17/19	28/30	17/19	<0.001
30 vs 19	*v_p_*	0.911	25/30	18/19	25/26	18/23	<0.001
	TTP	0.975	29/30	17/19	29/31	17/18	<0.001
	MAXConc	0.744	16/30	17/19	16/18	17/31	0.004
	AUC	0.747	19/30	15/19	19/23	15/26	0.004
	MAXSlope	0.853	22/30	17/19	22/24	17/25	<0.001

AUC of ROC, area under the curve receiver operating characteristic curve; NPV, negative predictive value; PPV, positive predictive value.

**Table 4:** Determination of independent risk factor in diagnosing breast cancers with different subtypes.

parameters	*P* value	OR value
TNBC vs. non-TNBC		
*k_ep_*	0.025	6.4
TTP	<0.001	13.6
HER2+ vs. HER2-		
TTP	0.013	5.9

With multi-variable regression analysis, *k_ep_* and TTP were revealed as independent risk factors in distinguishing TNBC from non-TNBC, and TTP was shown as the independent risk factor in distinguishing HER2 positive from HER2 negative.

## Discussion

In this study, we primarily explored the potential clinical application of DISCO imaging-derived semi-quantitative and quantitative perfusion parameters in diagnosing different types of breast cancer. The findings showed significant variances in semi-quantitative parameters such as TTP, MAX Conc, AUC, and MAX Slope between HER2-positive and HER2-negative groups, TNBC and non-TNBC groups, and HER2-positive and TNBC groups. Similarly, considerable differences were observed in the quantitative parameters of Ktrans, kep and vp among the aforementioned groups. Further, ROC analysis showcased the powerful diagnostic effectiveness of Ktrans, kep, vp, TTP, and MAX Conc in distinguishing between TNBC and non-TNBC and HER2-positive and HER2-negative. Furthermore, Ktrans, kep, vp, and TTP proved efficient in differentiating HER2-positive from TNBC. Notably, kep and TTP were pinpointed as independent risk factors for distinguishing HER2, while TTP stood out as an independent risk factor for distinguishing TNBC. This suggests that these parameters may serve as predictive imaging markers (26).

The semi-quantitative parameters of TTP, MAX Conc, AUC, and MAX Slope in our study showed significant differences between the HER2-positive and HER2-negative groups, TNBC and non-TNBC groups, and HER2-positive and TNBC groups. This suggests that the onset of the angiogenic “switch” might occur during hyperplasia and the most significant increase in angiogenesis might accompany the process of invasion. DCE-MRI perfusion parameters can potentially determine the success of anti-angiogenic therapy, treatment response or neo-adjuvant chemotherapy, and long-term survival outcomes. The correlation between mature blood vessels and the increase in HER2-positive tumor blood vessels was confirmed without interference from other factors (15). Prior studies have suggested a statistically positive correlation between the kep value and the expression of HER2. kep has been validated as an independent diagnostic factor for distinguishing HER2 molecular subtypes of breast cancer (16, 17), which aligns with our study’s results.

Contrary to previous studies (18, 19), our study did not find any differences in ve values between the HER2-positive and HER2-negative groups, TNBC and non-TNBC groups, or HER2-positive and TNBC groups. Nagasaka (17) reported a stronger correlation with HER2-positive breast cancer than Ktrans and kep. However, our study did not observe this relationship, potentially due to differences in ROI selection, variations in the pharmacokinetic analytic model, and the substantial heterogeneity of breast cancer. The discrepancy could also be influenced by the low incidence of HER2-positive cancers and bias in case selection (20). While the exact reason for the difference with previous results is uncertain, technical disparities in our calculation method and potential sampling bias might have affected our findings. More in-depth analyses are required to draw more reliable conclusions.

Our research focused on ultrafast DCE-MRI, specifically its connection to the characteristics of breast cancer via semi-quantitative and quantitative perfusion measures. While previous research (6) has established a connection between semi-quantitative parameters derived from ultrafast DCE-MRI and breast cancer features, there has been less exploration of quantitative perfusion parameters. In our investigation, we studied seven kinetic parameters generated from ultrafast DCE-MRI and found strong associations with HER2-positive and TNBC subtypes. Notably, we found significant variations in Ktrans, kep, vp, TTP, MAX Conc, AUC, and MAX Slope among the HER2-positive, HER2-negative, TNBC, and non-TNBC groups, indicating that these parameters could be utilized as distinct imaging indicators. While tissue biomarkers currently serve as the standard in managing breast cancer, predictive imaging markers can offer noninvasive insights into tumor biology, particularly valuable in scenarios where surgery or repeated biopsy isn’t optimal. Our study demonstrates that Ultrafast DCE MRI accurately distinguishes HER2-positive breast cancer and TNBC, avoiding invasive tests. Ultrafast DCE MRI parameters may serve as predictive imaging markers, aiding in understanding how tumor dynamics alter during neoadjuvant treatment and how these changes differ based on tumor heterogeneity. Consequently, Ultrafast DCE MRI predictive imaging markers warrant further research.

Despite the promising findings, our study had some limitations. It was a retrospective analysis that incorporated both diagnostic and screening procedures. The selection and outlining of regions of interest (ROI) were constrained by equipment capabilities, which may have introduced a subjective bias in lesion selection and potential overestimation of results. Additionally, while our cohort of 96 patients showed good correlation, larger multi-institutional studies with more diverse patient populations are warranted to draw more robust conclusions regarding the diagnostic utility of ultrafast DCE-MRI in breast cancer subtypes. Furthermore, while focusing on the overall HER2-positive group based on clinical standards, the emerging HER2 low subgroup warrants investigation via ultrafast DCE-MRI, as this subgroup with low HER2 expression levels may exhibit distinct biology and therapeutic responses compared to HER2-negative and HER2-positive subtypes (12, 21, 22). Evaluating ultrafast DCE-MRI’s ability to differentiate the HER2 low subgroup could provide insights for personalized treatment. Lastly, while this study focused on overall TNBC and HER2-positive subtypes, TNBC exhibits molecular heterogeneity with at least seven categories, each potential therapeutic targets (23, 24). Future work should explore ultrafast DCE-MRI’s capability in distinguishing TNBC molecular subtypes to gain insights for personalized treatment strategies (25).

## Conclusion

In conclusion, our study demonstrates a significant association between parameters obtained from ultrafast DCE-MRI and both HER2-positive breast cancer and TNBC. Although these results necessitate further validation via larger, multi-institutional studies, ultrafast DCE-MRI shows considerable potential. These findings underscore the promising role of ultrafast DISCO imaging and the markers derived from it in facilitating personalized treatment selection and monitoring therapeutic responses for breast cancer patients. The ability to provide valuable insights into changes in tumor dynamics during neoadjuvant treatment and variations among different tumor subtypes holds the potential to enhance the breast MRI protocol, offering crucial prognostic data and improving clinical outcomes in breast cancer management.

## Data Availability

The raw data supporting the conclusions of this article will be made available by the authors, without undue reservation.

## References

[1] Ramtohul T Tescher C Vaflard P Cyrta J Girard N Malhaire C. Prospective evaluation of ultrafast breast MRI for predicting pathologic response after neoadjuvant therapies. Radiology. (2022) 305(3):565–74. doi: 10.1148/radiol.220389 35880977

[2] Zhang S Rauch GM Adrada BE Boge M Mohamed RMM Abdelhafez AH. Assessment of early response to neoadjuvant systemic therapy in triple-negative breast cancer using amide proton transfer-weighted chemical exchange saturation transfer MRI: A pilot study. Radiol Imaging Cancer. (2021) 3:e200155. doi: 10.1148/rycan.2021200155 34477453 PMC8489465

[3] Gao W Zhang S Guo J Wei X Li X Diao Y. Investigation of synthetic relaxometry and diffusion measures in the differentiation of benign and Malignant breast lesions as compared to BI-RADS. J Magn Reson Imaging. (2021) 53:1118–27. doi: 10.1002/jmri.27435 33179809

[4] Wei Y Chen G Tang H Yuan Y Huang Z He X. Improved display of hepatic arterial anatomy using differential subsampling with cartesian ordering (DISCO) with gadoxetic acid-enhanced MRI: comparison with single arterial phase MRI and computed tomographic angiography. J Magn Reson Imaging. (2020) 51:1766–76. doi: 10.1002/jmri.27020 31837079

[5] Yau C Osdoit M van der Noordaa M Shad S Wei J de Croze D. Residual cancer burden after neoadjuvant chemotherapy and long-term survival outcomes in breast cancer: a multicentre pooled analysis of 5161 patients. Lancet Oncol. (2022) 23:149–60. doi: 10.1016/S1470-2045(21)00589-1 PMC9455620 34902335

[6] von Minckwitz G Huang CS Mano MS Loibl S Mamounas EP Untch M. T rastuzumab emtansine for residual invasive HER2 positive breast cancer. N Engl J Med. (2019) 380:617–28. doi: 10.1056/NEJMoa1814017 30516102

[7] Loi L Zimmermann F Goerke S Korzowski A Meissner JE Deike-Hofmann K. Relaxation-compensated CEST (chemical exchange saturation transfer) imaging in breast cancer diagnostics at 7T. Eur J Radiol. (2020) 129:109068. doi: 10.1016/j.ejrad.2020.109068 32574936

[8] Katayama A Miligy IM Shiino S Toss MS Eldib K Kurozumi S. Predictors of pathological complete response to neoadjuvant treatment and changes to post-neoadjuvant HER2 status in HER2-positive invasive breast cancer. Mod Pathol. (2021) 34:1271–81. doi: 10.1038/s41379-021-00738-5 PMC8216906 33526875

[9] Milon A Vande Perre S Poujol J Trop I Kermarrec E Bekhouche A. Abbreviated breast MRI combining FAST protocol and high temporal resolution (HTR) dynamic contrast enhanced (DCE) sequence. Eur J Radiol. (2019) 117:199–208. doi: 10.1016/j.ejrad.2019.06.022 31307648

[10] Herrmann KH Baltzer PA Dietzel M Krumbein I Geppert C Kaiser WA. Resolving arterial phase and temporal enhancement characteristics in DCE MRM at high spatial resolution with TWIST acquisition. J Magn Reson Imaging. (2011) 34:973–82. doi: 10.1002/jmri.22689 21769981

[11] Pelissier M Ambarki K Salleron J Henrot P. Maximum slope using ultrafast breast DCE-MRI at 1.5 Tesla: a potential tool for predicting breast lesion aggressiveness. Eur Radiol. (2021) 31:9556–66. doi: 10.1007/s00330-021-08089-0 34117556

[12] Shin SU Cho N Kim SY Lee SH Chang JM Moon WK. Time-to-enhancement at ultrafast breast DCE-MRI: potential imaging biomarker of tumour aggressiveness. Eur Radiol. (2020) 30:4058–68. doi: 10.1007/s00330-020-06693-0 32144456

[13] Donati F Boraschi P Cervelli R Pacciardi F Lombardo C Boggi U. 3 T MR perfusion of solid pancreatic lesions using dynamic contrast-enhanced DISCO sequence: Usefulness of qualitative and quantitative analyses in a pilot study. Magn Reson Imaging. (2019) 59:105–13. doi: 10.1016/j.mri.2019.03.001 30878601

[14] Koo TK Li MY. A guideline of selecting and reporting intraclass correlation coefficients for reliability research. J Chiropr Med. (2016) 15:155–63. doi: 10.1016/j.jcm.2016.02.012 PMC4913118 27330520

[15] Byon JH Park YV Yoon JH Moon HJ Kim EK Kim MJ. Added value of MRI for invasive breast cancer including the entire axilla for evaluation of high-level or advanced axillary lymph node metastasis in the post–ACOSOG Z0011 trial era. Radiology. (2021) 300:46–54. doi: 10.1148/radiol.2021202683 33904772

[16] Du S Gao S Zhang L Yang X Qi X Li S. Improved discrimination of molecular subtypes in invasive breast cancer: Comparison of multiple quantitative parameters from breast MRI. Magnetic Resonance Imaging. (2021) 77:148–58. doi: 10.1016/j.mri.2020.12.001 33309922

[17] Zhou X Gao F Duan S Zhang L Liu Y Zhou J. Radiomic features of Pk-DCE MRI parameters based on the extensive Tofts model in application of breast cancer. Australas Phys Eng Sci Med. (2020) 43:517–24. doi: 10.1007/s13246-020-00852-9 32524436

[18] Nagasaka K Satake H Ishigaki S Kawai H Naganawa S. Histogram analysis of quantitative pharmacokinetic parameters on DCE-MRI: correlations with prognostic factors and molecular subtypes in breast cancer. Breast Cancer. (2019) 26:113–24. doi: 10.1007/s12282-018-0899-8 30069785

[19] Choi EJ Choi H Choi SA Youk JH. Dynamic contrast-enhanced breast magnetic resonance imaging for the prediction of early and late recurrences in breast cancer. Medicine. (2016) 95. doi: 10.1097/MD.0000000000005330 PMC5134812 27902592

[20] Brunetti B Bacci B Angeli C Benazzi C Muscatello LV. P53, ER, and ki67 expression in canine mammary carcinomas and correlation with pathological variables and prognosis. Veterinary Pathol. (2021) 58:325–31. doi: 10.1177/0300985820973462 33208018

[21] Heacock L Lewin A Ayoola A Moccaldi M Babb JS Kim SG. Dynamic contrast-enhanced MRI evaluation of pathologic complete response in human epidermal growth factor receptor 2 (HER2)-positive breast cancer after HER2-targeted therapy. Acad Radiol. (2020) 27:e87–93. doi: 10.1016/j.acra.2019.07.011 PMC7416507 31444111

[22] Chen P Zhao S Guo W Shao G. Dynamic contrast-enhanced magnetic resonance imaging features and apparent diffusion coefficient value of HER2-positive/HR-negative breast carcinoma. Quant Imaging Med Surg. (2023) 13:4816–25. doi: 10.21037/qims-22-1318 PMC10423352 37581065

[23] Lee YM Oh MH Go JH Han K Choi SY. Molecular subtypes of triple-negative breast cancer: understanding of subtype categories and clinical implication. Genes Genomics. (2020) 42:1381–7. doi: 10.1007/s13258-020-01014-7 33145728

[24] Zhao Y Huang T Jin X Gong XM Lu YZ. Clinicopathologic features and immune cell subtypes analysis of tumor-infiltrating lymphocytes rich invasive breast carcinoma of no special type. Appl Immunohistochem Mol Morphol. (2023) 31:354–62. doi: 10.1097/PAI.0000000000001125 PMC10321512 37278279

[25] Guo L Xie G Wang R Yang L Sun L Xu M. Local treatment for triple-negative breast cancer patients undergoing chemotherapy: breast-conserving surgery or total mastectomy? BMC Cancer. (2021) 21:717. doi: 10.1186/s12885-021-08429-9 34147061 PMC8214797

[26] Kang SR Kim HW Kim HS. Evaluating the relationship between dynamic contrast-enhanced MRI (DCE-MRI) parameters and pathological characteristics in breast cancer. J Magnetic Resonance Imaging. (2020) 52:1360–73. doi: 10.1002/jmri.27241 32524658

